# Evaluation of a Mobile Health App Offering Fertility Information to Male Patients With Cancer: Usability Study

**DOI:** 10.2196/33594

**Published:** 2022-05-04

**Authors:** Eden Noah Gelgoot, Katya Kruglova, Peter Chan, Kirk Lo, Zeev Rosberger, Philippa Chown, Jordana Kazdan, Siobhan Bernadette Laura O’Connell, Phyllis Zelkowitz

**Affiliations:** 1 Lady Davis Institute for Medical Research Jewish General Hospital Montréal, QC Canada; 2 Department of Psychiatry Jewish General Hospital Montréal, QC Canada; 3 McGill University Health Centre Montréal, QC Canada; 4 Department of Surgery McGill University Montréal, QC Canada; 5 Department of Surgery Mount Sinai Hospital Toronto, ON Canada; 6 Department of Surgery University of Toronto Toronto, ON Canada; 7 Department of Psychiatry McGill University Montréal, QC Canada; 8 Department of Psychology McGill University Montréal, QC Canada; 9 Department of Oncology McGill University Montréal, QC Canada

**Keywords:** mobile app, eHealth, male, cancer, infertility, fertility preservation, psycho-oncology

## Abstract

**Background:**

Cancer and its treatment can adversely affect male fertility. Although sperm banking is an effective fertility preservation method, there is an unmet need for information and support surrounding these issues.

**Objective:**

This usability study evaluates a mobile health app providing male patients with cancer with credible information about the impact of cancer and its treatment on fertility and fertility preservation.

**Methods:**

Participants were recruited by a market research firm. Eligibility criteria were men who were 18-45 years of age, identified as male, diagnosed with new or recurring cancer within 1 year, not in fertility treatment, able to read and write in English or French, and had internet access. App usage was tracked for 2 weeks. After app use, participants provided qualitative feedback about their experiences using the app as well as quantitative data regarding their sperm banking decisions, perceived change in fertility knowledge, evaluation of the app’s information on the Information Assessment Method, and the app’s quality on the user version of the Mobile App Rating Scale.

**Results:**

The sample included 40 men aged 27-45 years. Approximately 68% (27/40) indicated that no one had previously spoken to them about the impact of cancer on fertility, and 85% (34/40) had not received information on fertility preservation. Approximately 83% (33/40) found the app’s information relevant, and 85% (34/40) said that it increased their fertility knowledge. Approximately 23% (9/40) made a decision about sperm banking after using the app. Participants rated the app’s quality highly, with mean scores (out of 5) of 4.14 for information, 4.06 for functionality, 3.84 for aesthetics, and 3.63 for engagement.

**Conclusions:**

The app proved to be useful for male patients with cancer, suggesting that mobile health resources could be beneficial to incorporate into clinical care to enable shared decision-making about fertility.

## Introduction

About 8600 Canadian boys and men aged 15-39 years are diagnosed with cancer yearly [1]. Cancer can adversely affect male fertility by damaging the reproductive organs, disrupting hormone levels, or impairing sperm production/release [2]. Male fertility can also be affected by cancer treatment, including chemotherapy, radiation, and surgery [3-6]. As survival rates improve [7], patients face long-term consequences of cancer and its treatment [8]. Psychological distress is common among men with cancer who may fear disease recurrence or feel inadequate and for whom cancer might interfere with career goals and family planning [9]. Cancer treatment may result in decreased libido, sexual dissatisfaction, erectile dysfunction [9], and cause difficulties in cultivating intimate relationships [10].

The most established method to preserve male fertility before cancer treatment is semen cryopreservation, also known as sperm banking [4,11]. For most patients, the semen sample is collected via masturbation [12]. However, in patients with difficulties providing a semen sample via ejaculation, there are a variety of alternative sperm retrieval techniques that can be used (eg, electroejaculation, aspiration of sperm from the testicle or epididymis) [12,13]. Banked sperm can then be used to achieve a pregnancy with the use of assisted reproductive techniques such as in vitro fertilization and intracytoplasmic sperm injection [4]. There are also options for men who do not have viable sperm, such as the use of donor sperm in conjunction with in vitro fertilization and intracytoplasmic sperm injection, as well as adoption [11].

Although sperm banking is an effective fertility preservation method [14], there is an unmet need for information and support surrounding these issues [15-17]. Most male patients with cancer view receiving fertility information as very important but are often dissatisfied with the information obtained [16]. The urgency to begin treatment or fear of passing cancer to offspring may act as barriers to sperm banking [15,18,19]. Factors that often prevent fertility preservation conversations include the potential distress from discussing infertility risk, limited access to educational materials, and clinicians’ lack of time and knowledge [20]. Additionally, men may not initiate these conversations since they are generally less likely than women to ask questions during medical appointments [21].

There is a need for fertility preservation resources to be better integrated into cancer care [4,15,18,22]. In a survey conducted by our team, 80% of male patients with cancer preferred receiving fertility information at the first oncology consult or at the time of diagnosis and treatment planning [15]. Loren et al [18] recommend that referrals to counselling services be incorporated into routine care for men with fertility concerns. Thus, it is imperative that clinicians discuss fertility preservation with patients as early as possible and refer them to reproductive specialists.

eHealth resources are viewed positively by cancer survivors [23] and are suitable for men who often value autonomy and anonymity when seeking information [24]. However, current web-based information for male patients with cancer is not comprehensive, less accessible than that for female patients [25], of inadequate readability and quality [26], and is not rigorously evaluated [27]. One study has assessed the feasibility of a web-based intervention targeting fertility distress after cancer, but their sample includes only 4 men [28,29]. Given the widespread use of smartphones [30], mobile health (mHealth) apps show promise as tools to improve the quality of life of patients with cancer [31].

To address the need for fertility information tailored to male patients with cancer, our team developed an mHealth app, *Infotility XY*, providing information on the impact of cancer and its treatment on male fertility and fertility preservation. In this study, we evaluate the app’s quality and information, as well as its potential to improve fertility knowledge and help patients make fertility preservation decisions.

## Methods

### App Study Design

The study design for the *Infotility XY* app adhered to the Medical Research Council guidelines for the development and evaluation of complex interventions [32]. The guidelines include 4 phases: development, feasibility and piloting, evaluation, and implementation [32].

In the development phase of the study, our team designed 3 versions of the *Infotility/Infotilité XY* app for 3 populations in collaboration with an app development company: men in the general public, male patients with infertility, and male patients with cancer. In this paper, only data from the sample of patients with cancer are presented.

The app content was written by our team and informed by extensive literature reviews and a needs assessment survey of the fertility-related informational and support needs of male patients with cancer [15]*.* Key stakeholders, including male patients with cancer, were included throughout the app development process, informing the app’s content and design elements. Content was vetted by health professionals and experts in patient-centered care. All content was available in English and French.

In addition to information about sperm banking, the app for male patients with cancer provided information on fertility treatment in general (eg, in vitro fertilization) as well as the use of donor sperm. The app also addressed common concerns among male patients with cancer such as the risk of passing their cancer onto future children, which was a concern that came up in the needs assessment survey among male patients with cancer. The app included 19 articles grouped into 4 categories: “Fertility and cancer,” “Sperm banking 101,” “After banking,” and “Talking to my partner about sperm banking” (see Multimedia Appendix 1 for the list of the articles). Each article had the option to give a thumbs-up/down to indicate its usefulness. The app featured pop-up glossary definitions, infographics, animations, and a Canada-wide map of fertility clinics (see Multimedia Appendix 2 for screenshots of the app).

Our research team collaborated with an app company to transform the informational content into a user-friendly app. The app company helped develop the look and feel of the app (eg, color scheme, graphics), the different features in the app (eg, map of fertility clinics, pop-up glossary definitions), and the navigation. The app company did not have access to users’ data.

In the feasibility/piloting phase of the study, an interactive prototype of the *Infotility XY* app was developed, which allowed the research team to make changes to the organization of the information before presenting the app to participants.

In the evaluation phase of the study, we assessed the uptake and usability of the app by using a pre-post study design. We determined our 2 main outcome measures (the user version of the Mobile App Rating Scale [uMARS] and the Information Assessment Method [IAM]) based on literature reviews of available tools to assess the quality and information of apps. The next phase of the study is implementation, which includes finding partners to disseminate the app and provide long-term follow-up and monitoring [32].

### Ethics Approval and Recruitment

This study was approved by the Medical/Biomedical Research Ethics Committee of CIUSSS (Centre intégré universitaire de santé et de services sociaux) West-Central Montreal Research Ethics Board (MP-05-2016-344). Participants were recruited between August and October 2020 across Canada by a market research firm (“recruitment company”). The recruitment company was selected based on their experience in medical research, their ability to recruit a representative sample of participants from Canada, and their adherence to the highest standards in research methodology, ethical practices, respondent rights, and personal privacy. The recruitment company did not have access to participants’ data. In the communications between our team and the recruitment company, participants were referred to by their unique code, which did not identify them, to protect participants’ confidentiality. The recruitment company recruited patients with cancer via physicians and patient advocacy groups and contacted them via email and telephone. The recruitment company screened potential participants for the following criteria: identified as male, had internet access, able to read and write in English/French, aged 18-45 years, diagnosed with new/recurrent cancer within the past year, and not in fertility treatment. Individuals who met the eligibility criteria and provided written informed consent were enrolled in the study. Once the target sample of 40 participants was reached, recruitment was terminated.

### Participants

Guidelines for this phase of the evaluation of web-based interventions suggest that a sample of at least 20 users is required for statistical significance [33]. To account for possible attrition, we aimed to recruit 40 men. The recruitment company contacted 586 patients with cancer; 63 agreed to be screened, 43 were eligible and consented, and 40 completed the study. Of these 40 men, 24 were recruited via referrals from health care providers, 11 via patient referrals, and 5 via the recruitment company’s database.

### Procedures

After providing informed consent online, participants created an app account, completed pre–app usage questionnaires, and gained access to the app for 2 weeks. This period was selected based on our previous experience [34], where app usage tended to drop off after 2 weeks. After app use, participants were blocked from viewing the app and directed to post–app usage questionnaires. After completing the questionnaires, participants regained app access. To reduce attrition, participants were sent up to 3 reminder emails to complete questionnaires and use the app. Participants received CAD $150 from the recruitment company upon study completion. See Multimedia Appendix 3 for the study’s procedures.

### Measures

#### Background Questionnaire

Participants provided information about their sociodemographic characteristics, including relationship status, age, ethnicity, immigrant status, education, income, religion, and parity. Participants were also asked whether anyone had spoken to them about the impact of cancer on fertility, whether they received information about fertility preservation, and if so, whether they received all the information they needed, their most recent cancer diagnosis, and the age at which they received it, and their current cancer status.

#### Fertility Knowledge and Preservation

After app use, participants were asked (1) whether the app increased their knowledge of fertility in relation to cancer, using a scale from 0 (“No, not at all”) to 3 (“Yes, quite a lot”); (2) whether they made a decision about sperm banking during the study (yes/no); and (3) if they selected “yes,” they were asked what decision they made (eg, I banked my sperm), and what factors helped them make the decision.

#### IAM

The IAM was used to evaluate participants’ ratings of the app’s information. The measure was developed to assess the relevance, cognitive impact, use, and health benefits of web-based health information and has been validated with patients and consumers of web-based health information [35,36]. Our team adapted the 8-item measure from the 2019 IAM version for Fertility and the IAM4All. All items are considered individually. No total scores or cutoffs exist.

#### uMARS

The uMARS was used to measure participants’ rating of the app’s quality. This 20-item measure consists of 4 subscales. The Engagement subscale measures whether the app is interesting, customizable, and interactive; the Functionality subscale asks about the app’s functionality and navigation; the Aesthetics subscale asks about the app’s visual appeal; and the Information subscale asks whether the app contains credible, high quality information. Each subscale is measured on a scale from 1-5; higher scores represent higher ratings. The mean score is obtained by averaging the 4 subscales’ scores. An additional 4 items measuring the app’s subjective quality can be averaged to obtain a *subjective* quality score. The uMARS was developed by Stoyanov et al [37] and tested in a sample of Australians aged 16-25 years. The Flesch-Kincaid readability test indicated that the uMARS required a grade 8 reading level [37]. The total score demonstrated excellent internal consistency (α=.90) and interrater reliability (intraclass correlation=0.79) [38]. Each subscale demonstrated satisfactory consistency, with Cronbach alpha ranging from .70 to .80 [37].

#### Qualitative and Quantitative Data on App Usage

To capture participants’ experiences using the app, our team developed open-ended questions.

1. Please describe any fertility topics or features that were not included in the app and that you would have liked to be included. Please tell us why you want those topics or features to be included.
2. Please tell us what you liked best about the app and why.
3. Please tell us what you liked least about the app and why.

We present quantitative data for the following app usage metrics: unique pageviews and thumbs-up/down assessments.

#### Quantitative Analyses

No questionnaire data were missing. Quantitative analyses were performed using SPSS (IBM Corp). Descriptive quantitative analyses were used to assess participants’ sociodemographic characteristics and informational needs, the influence of the app on treatment decisions and fertility knowledge, and evaluation of the app’s information and quality. Given the small sample size (N=40), we did not conduct multivariate analyses. However, descriptive statistics were sufficient in answering our overarching question regarding the usability of the app in conjunction with the qualitative feedback.

#### App Usage

The app company compiled the app usage metrics. For each participant, the numbers of unique pages viewed and thumbs-up/down assessments were extracted. These metrics were presented as totals and were also classified into categories: medical (11 articles), legal (3 articles), or psychosocial (5 articles; Multimedia Appendix 1). Developed for analytic purposes, these categories were not seen by participants. If a participant visited a page multiple times, it was only counted once. No app usage data were missing.

#### Qualitative Feedback

All participants responded to the open-ended questions assessed in the questionnaires delivered after using the app. Their feedback was analyzed by 2 researchers (KK and ENG) on a qualitative data analysis software (NVivo, QSR International) using directed content analysis with an iterative approach [39]. A directed content analysis approach allows researchers to use predetermined codes. The uMARS dimensions of aesthetics, functionality, engagement, and information guided analyses and were used as the pre-existing codes. These categories allowed researchers to understand participants’ qualitative feedback in relation to the quantitative data, which also looked at users’ perceptions of the app on these quality rating scales. After the first round of coding, discrepancies were discussed and resolved between 2 researchers.

## Results

### Sociodemographic Data

The sample consisted of 40 patients with cancer, all of whom accessed the app in English (see Multimedia Appendix 4 for sociodemographics). The age range was 27-45 years (mean 36.93 [SD 5.48] years). Most participants were in heterosexual relationships (27/40, 68%), followed by single (8/40, 20%), and in nonheterosexual relationships (5/40, 13%). More than half of the men had children (22/40, 55%), and most indicated that they would like to have children in the future (33/40, 83%). Most were White (25/40, 63%), born in Canada (35/40, 88%), had an income between CAD $50,000-CAD $89,999 (19/40, 48%), had a high school or CEGEP (Collège d'enseignement général et professionnel) education level (23/40, 58%), and were not religious (25/40, 63%). During the study, approximately 68% (27/40) of the participants were in cancer treatment, 25% (10/40) in partial remission, and 8% (3/40) in remission, with an average remission time of 1 year (SD 1.73, range 0-3). The most common diagnoses were prostate cancer (7/40, 18%), testicular cancer (7/40, 18%), skin cancer (5/40, 13%), and bladder cancer (4/40, 10%). The average age of diagnosis was 36.1 (SD 5.49) years (range 26-45 years).

### Information Seeking

Of the 40 participants, 27 (68%) indicated that no one had ever spoken to them about the impact of cancer on fertility and 34 (85%) had not received information on fertility preservation. Of those who did receive this information, 67% (4/6) did not get all the information they needed.

### App Usage

On average, participants viewed 99% (18.80/19) of the app’s articles (SD 0.97, range 13-19), and specifically 99% of the medical articles (10.93/11, SD 0.27, range 10-11), and 98% of psychosocial articles (4.88/5, SD 0.79, range 0-5). All participants viewed each of the 3 lifestyle articles. Participants gave a thumbs-up to an average of 7.85 (SD 7.94, range 0-19) articles and specifically to an average of 4.53 (SD 4.59, range 0-11) medical articles, 1.40 (SD 1.39, range 0-3) lifestyle articles, and 1.93 (SD 2.24, range 0-5) psychosocial articles. No article received a thumbs-down.

### Fertility Knowledge and Preservation

Of the 40 participants, 34 (85%) said the app increased their fertility knowledge. Prior to the study, 95% (38/40) of men had not banked their sperm. During the study, 23% (9/40) of the participants made a decision about sperm banking: 1 decided to bank his sperm, 7 are planning to do so in the future, and 1 decided not to. Of the 8 who decided to bank their sperm, 6 (75%) said the app helped them make the decision.

### Evaluation of the App’s Information

80% (32/40) of the participants viewed the app to satisfy their curiosity about a health matter (Table 1). Approximately 83% (33/40) found the information relevant, 95% (38/40) understood the information well, and 83% (33/40) learned something new. Of the 78% (31/40) who used the information for themselves, 90% (28/31) said the information helped them better understand a particular health issue. Of the 85% (34/40) who benefited (or expect to benefit) from the information, 79% (27/34) said the information helped them feel less worried about a health problem and 53% (18/34) said it facilitated their communication with health professionals.

**Table 1:** Data on the app’s information evaluated by the Information Assessment Method (IAM) (N=40).

Information Assessment Method question		Values, n (%)
**Why did you look on this app for information?**		
	To answer a question about my health	27 (68)
	To answer a question about the health of someone else	12 (30)
	To satisfy my curiosity about a health matter	32 (80)
	To help me decide if I should see a health professional	13 (33)
	To prepare myself before talking to a health professional	8 (20)
	To follow up on the information given by a health professional	5 (13)
	To find choices different from those given by a health professional	6 (12)
**Is the app’s information relevant?**		
	Very little relevant	3 (8)
	Somewhat relevant	4 (10)
	Relevant	19 (48)
	Very relevant	14 (35)
**Did you understand the app’s information? **		
	Very poorly	0
	Poorly	2 (5)
	Well	23 (58)
	Very well	15 (38)
**What do you think about the app’s information?**		
	Now I know something new	33 (83)
	This information says I did or I am doing the right thing	21 (53)
	Now I am reassured	22 (55)
	I am reminded of something I already knew	10 (25)
	Now I want to learn more about this health matter	16 (40)
	I am not satisfied with this information	3 (8)
	I think there is a problem with this information	0
	I think this information could be harmful	0
**Did you or will you use the app’s information for yourself? **		
	Yes	31 (78)
	No, not for myself, but I used this information for someone else	6 (15)
	No, I did not use this information for myself or for someone else	3 (8)
**If yes, how did you or will you use it?**		
	This information helped (will help) me to better understand a particular issue about my health.	28 (90)
	I did not know what to do, and this information helped (will help) me make a decision about my health.	16 (52)
	I knew what to do, and I used (will use) this information to be more certain about my health care.	12 (39)
	I was doing (going to do) something concerning my health, and I used (will use) this information to do it differently.	4 (13)
	I used (will use) this information in a discussion with a health professional	2 (7)
**Did you (do you expect to) benefit from the app’s information?**		
	Yes	34 (85)
	No	6 (15)
**If yes, how did you (do you expect to) benefit?**		
	This information helped (helps) me feel less worried about a health problem	27 (79)
	This information made (makes) me more satisfied with health care I receive	16 (40)
	This information allowed (will allow) me to better communicate with a health professional	18 (53)
	Because of this information, I was (will be) more involved in decisions about my health	14 (41)
	This information helped (will help) me to better handle a problem with my health	9 (27)
	This information helped (will help) me prevent a health problem or the worsening of a health problem	2 (6)
	This information helped (will help) to improve my health	1 (3)

### Evaluation of the App’s Quality

Participants rated the app’s quality highly (Table 2). The average quality rating was the highest for information, followed by functionality. The lowest rated subscale was engagement, though it was still rated 3.63/5.00 on average. Most men would recommend the app.

**Table 2:** App quality analysis using the user version of the Mobile App Rating Scale (uMARS) (N=40).

uMARS item		Value
**Objective quality subscale, mean (SD)**		
	Engagement (range 2.20-4.80)	3.63 (0.75)
	Functionality (range 2.25-5.00)	4.06 (0.74)
	Aesthetics (range 2.67-5.00)	3.84 (0.65)
	Information (range 3.00-5.00)	4.14 (0.61)
	Objective quality total score (range 3.02-4.84)	3.92 (0.62)
What is your overall (star) rating of the app? (range 2.00-5.00), mean (SD)		3.75 (0.54)
**App rating, n (%)**		
	1 (One of the worst apps I’ve used)	0
	2	1 (3)
	3 (Average)	9 (23)
	4	29 (73)
	5 (One of the best apps I’ve used)	1 (3)
**Would you recommend this app to people who might benefit from it? n (%)**		
	Not at all	0
	Very few people	3 (8)
	Maybe	12 (30)
	Many people	17 (43)
	Definitely	8 (20)
**How many times do you think you would use this app in the next 12 months? n (%)**		
	None	3 (8)
	1-2	7 (18)
	3-10	20 (50)
	10-50	9 (23)
	>50	1 (3)
**Would you pay for this app? n (%)**		
	1 (Definitely not)	7 (18)
	2	8 (20)
	3	15 (38)
	4	8 (20)
	5 (Definitely yes)	2 (5)
Subjective quality total score (range 2.00-4.75), mean (SD)		3.30 (0.696)

### Qualitative Feedback

#### Engagement

Participants liked the videos because they were “interesting” (participant #24) and “informative” (participant #14), and they suggested including more videos. Men would have also liked the ability to connect with others, for example, to obtain “…feedback from people who have banked sperm…” (participant #4).

#### Functionality

Participants liked the app’s functionality, finding it “extremely easy to use and navigate” (participant #8) and that it had a “very intuitive design” (participant #9). Apart from being “neatly organized” (participant #29), men appreciated that the app allowed the user to “read at [his] own pace” (participant #29).

#### Information

Participants found the app “very educational and very useful” (participant #36) and appreciated that it was a “one stop shop for fertility info” (participant #23), which helped prevent information overload: “The link to detailed information is available on demand, it prevents from unnecessary information burden…” (participant #38). Participants liked that the information was “very comprehensive” (participant #26) and “… [was] applicable for different scenarios” (participant #39). However, some thought there was “too much information” (participant #22).

Participants appreciated that the app included “a lot of good links and honest information about [w]here to go for help” (participant #19). They particularly liked the sperm banking resources, saying that the app “help[ed] locat[e] sperm banks near me” (participant #5). Participants wanted “more cost-based information” (participant #16), including the “average cost of each procedure” (participant #27) and “if [each procedure is] covered by health care…” (participant #19). Participants also wanted more in-depth information about the effects of cancer on fertility, for example, about “… certain types of cancers and how it affects each one differently” (participant #10).

Participants valued that the app had a “wealth of useful info from very trustworthy sources” (participant #17). They also thought the information “was very easy to read” (participant #11), and “not too complicated or jargon heavy” (participant #7). However, 1 man would have liked if the information was “less wordy” (participant #30).

The app’s information made participants feel “reassured” (participant #25): “This app really made me feel comfortable about how I was feeling about my diagnosis and how to go about my family’s future” (participant #2). Men also mentioned that the information “ma[de] [him] feel safe and confident to look at donating sperm and how to do it” (participant #36). Though some found the information “depressing at times” (participant #6), overall men appreciated the “very supportive tone” of the app (participant #31).

## Discussion

### Principal Findings

Overall, participants valued *Infotility XY* as a source of comprehensive, relevant, and accessible information. Most participants had not received information about the impact of cancer on fertility or fertility preservation prior to the study. Those who did receive this information did not receive all the information they needed. After app use, most men felt that their fertility knowledge increased and that the information promoted better communication with clinicians, indicating that an mHealth app may be useful in clinical practice to address the fertility-related informational needs of male patients with cancer. Providing patients with written information may help initiate fertility discussions with medical staff, leading to a referral to a reproductive specialist [40].

The fact that most participants had not received fertility information prior to the study might have contributed to the high engagement level. Men seemed to be motivated to learn about fertility and sperm banking. Most participants found the information relevant, credible, and easy-to-read. Given the lack of oncofertility educational materials suitable for patients with varying health literacy levels [41], our study highlights the possibility of presenting scientific content in simple terms that is accessible to diverse patient groups.

Furthermore, although almost all men had never banked sperm prior to the intervention, 8 decided to bank during the study. Owing to lack of information, patients with cancer may not fully participate in decision-making regarding their future fertility, which can prevent them from banking sperm [42]. Our results indicate that an mHealth app can empower patients to feel more in control of their reproductive health and be proactive in preserving fertility. Furthermore, the information helped participants feel comforted and reassured that they were making the right decisions about their fertility. Thus, our study demonstrates the potential of an mHealth app to help address the fertility concerns of patients with cancer by providing evidence-based information in a supportive manner. Additionally, based on participants’ feedback, future mHealth apps should present a significant proportion of content in video format to help users with different health literacy levels understand and retain the material. A chat option may also benefit patients by allowing them to seek social support [43].

### Study Limitations and Strengths

This study has several limitations. First, there may have been selection bias since participants volunteered to enroll in the study. Thus, our sample may not fully reflect the broader population of male patients with cancer. As we remunerated participants in appreciation of their involvement in the research, they may have felt more inclined to complete the study or provide more positive feedback about the app, which may have introduced bias into our results. Second, since our sample was small and did not include Francophones, French content was not evaluated, potentially limiting the generalizability of results. Third, our sample did not include men aged 18-26 years. This subgroup might not be concerned with family building yet but should nevertheless be informed about the impact of cancer on fertility, and thus, it is an important group to include in future research.

Despite these limitations, our study has notable strengths. We used quantitative methods and content analysis, allowing for a nuanced understanding of participants’ experiences using the app. Our sample was socioeconomically diverse with respect to income and education. There was also variation in participants’ relationship and fatherhood statuses, suggesting generalizability of results to patients at different life stages. Recruiting people at the hospital bedside who were in active cancer treatment for a psychosocial research project may have been challenging, especially given that recruitment took place during the COVID-19 pandemic. Therefore, using a recruitment company who could recruit participants remotely allowed for us to successfully recruit our target sample size (N=40).

### Conclusions

This usability study provides preliminary support that an mHealth app may be valuable in clinical practice by assisting in educating patients about the impact of cancer on fertility, thereby helping them make fertility preservation decisions and providing comfort. To our knowledge, this study is the first to evaluate an mHealth app providing male patients with cancer with evidence-based information about the impact of cancer on fertility and fertility preservation. We are in contact with professional organizations and patient advocacy groups to engage in knowledge transfer and to plan future studies. Randomized controlled trials with larger samples are warranted to assess the effectiveness of mHealth interventions in improving patients’ fertility knowledge and influencing their sperm banking decisions. Further efforts are needed to increase the availability of evidence-based mHealth apps for patients with cancer.

## Appendix

Multimedia Appendix 1 Categories and articles of the Infotility XY app.

Multimedia Appendix 2 Design and features of the Infotility XY app.

Multimedia Appendix 3 Study procedures.

Multimedia Appendix 4 Sociodemographic characteristics of our sample.

## Abbreviations

IAM: Information Assessment Method
mHealth: mobile health
uMARS: user version of the Mobile App Rating Scale
