# Supplementary material for: Evaluation of a Mobile Health App Offering Fertility Information to Male Patients With Cancer: Usability Study
Source: JMIR Cancer. 2022 May 4;8(2):e33594. doi: 10.2196/33594 (PMC9118008; doi:10.2196/33594)
Supplement: Multimedia Appendix 1 [file cancer_v8i2e33594_app1.docx]

**Appendix 1. Categories and articles of the *Infotility XY* app**

| **Categories (as displayed on the app)** | **Articles** | **Description of article** | **Categories (for analytic purposes)** |
| --- | --- | --- | --- |
| **1. Fertility and cancer** | 1. How does cancer and its treatment affect fertility? | Effect of cancer and its treatment on male fertility | Medical |
|  | 1. All about sperm | Semen; sperm: parameters, DNA fragmentation | Medical |
|  | 1. Why should I bank my sperm? | Benefits and health risks of sperm banking | Medical |
|  | 1. I've heard that I can pass on cancer to my future child – is that true? | The risks of passing down cancer | Medical |
| **2. Sperm banking 101** | 1. What is sperm banking? | General information about cryopreservation | Medical |
|  | 1. Where should I go and how much will banking cost? | Referral for sperm banking; provincial coverage | Legal |
|  | 1. I found a sperm bank, now what? | Making an appointment at a fertility clinic; testing before sperm banking; consent form | Legal |
|  | 1. Providing a sample | Basics of providing a sperm sample; sperm evaluation; cryopreservation | Medical |
|  | 1. What if I can’t provide a sperm sample? | Sperm retrieval procedures | Medical |
|  | 1. More information on sperm storage | Long term storage of banked sperm: logistics, cost, removal | Legal |
|  | 1. I’m undecided about banking and need more information | Counselling; religious considerations; different options for having a family; sperm banking as an insurance policy | Medical |
|  | 1. Can I still bank if I already started my cancer treatment? | Things to consider if one has already started cancer treatment | Medical |
| **3. After banking** | 1. What do I do when I'm ready to use my sperm? | Semen analysis; fertility treatment; surrogacy and egg donation | Medical |
|  | 1. Assisted Reproductive Technology (ART) | Intrauterine insemination; in vitro fertilization; donor sperm, eggs and embryos; potential risks to offspring | Medical |
| **4. Talking to my partner about sperm banking** | 1. We've talked about having kids, but I'm unsure what to say | Tips for communication with a partner about sperm banking in different life situations | Psychosocial |
|  | 1. We've been dating for a while, but we haven't really talked about the future |  | Psychosocial |
|  | 1. We've just met. What should I say? |  | Psychosocial |
|  | 1. I am in a same-sex relationship. Should I still bank my sperm? |  | Psychosocial |
|  | 1. Talking about what happens to my frozen sperm |  | Psychosocial |
