# Supplementary material for: Evaluation of a Mobile Health App Offering Fertility Information to Male Patients With Cancer: Usability Study
Source: JMIR Cancer. 2022 May 4;8(2):e33594. doi: 10.2196/33594 (PMC9118008; doi:10.2196/33594)
Supplement: Multimedia Appendix 2 [file cancer_v8i2e33594_app2.docx]

**Appendix 2. Design and features of the *Infotility XY* app**


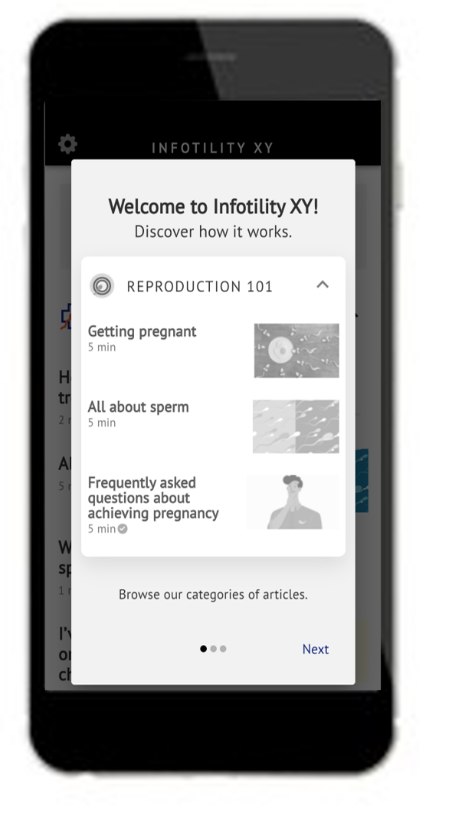

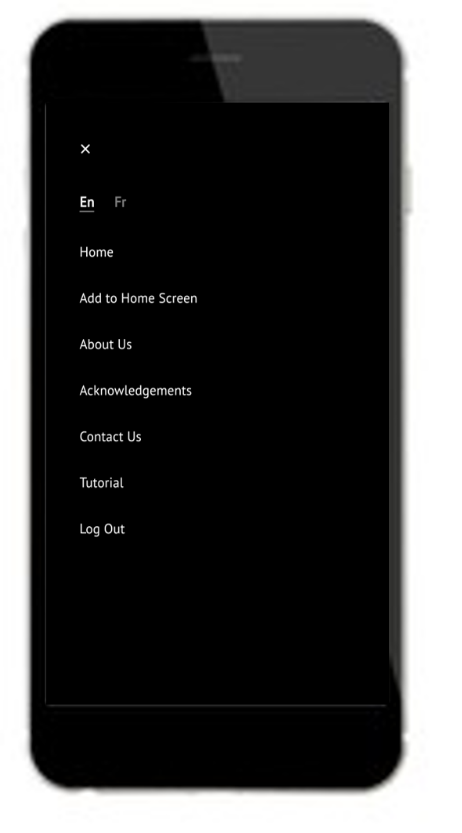


(A) (B)

**Figure S1.** The Tutorial (A) and the Settings menu (B).


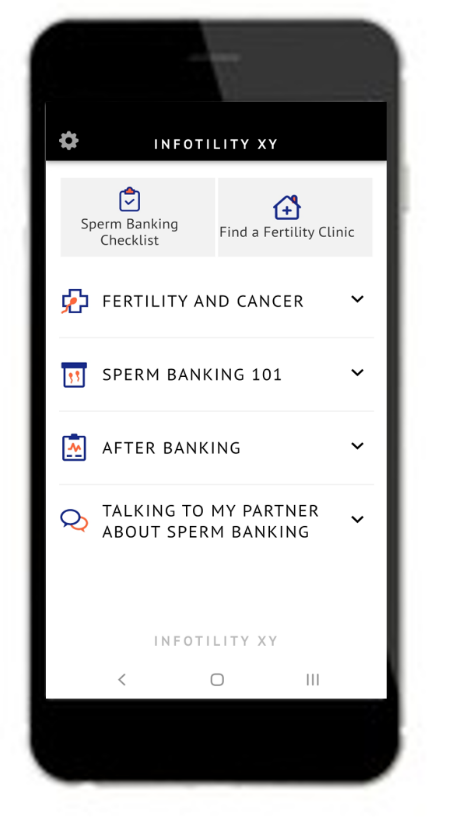

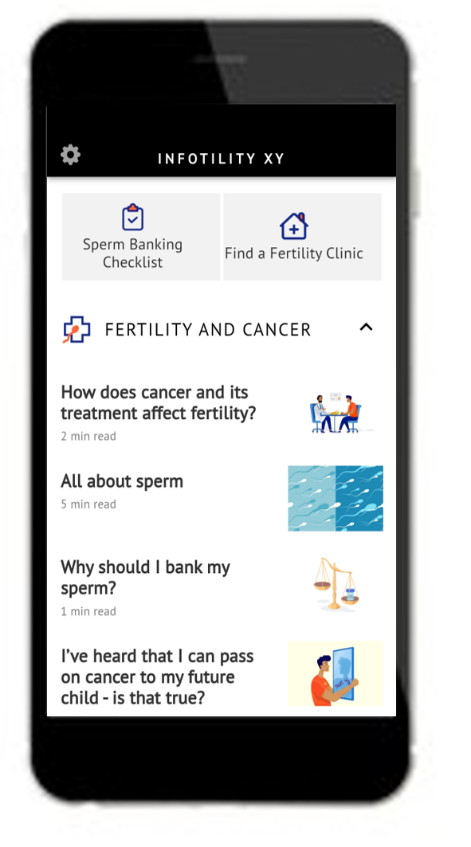


(A) (B)

**Figure S2.** The Home page with the article categories collapsed (A) and expanded (B).


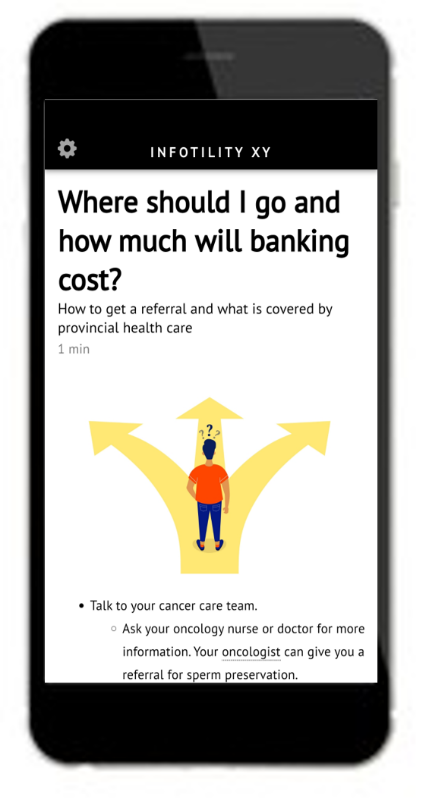

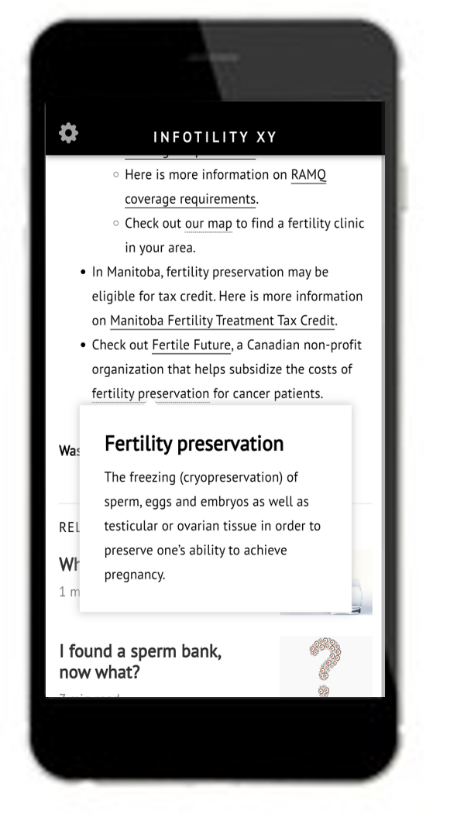

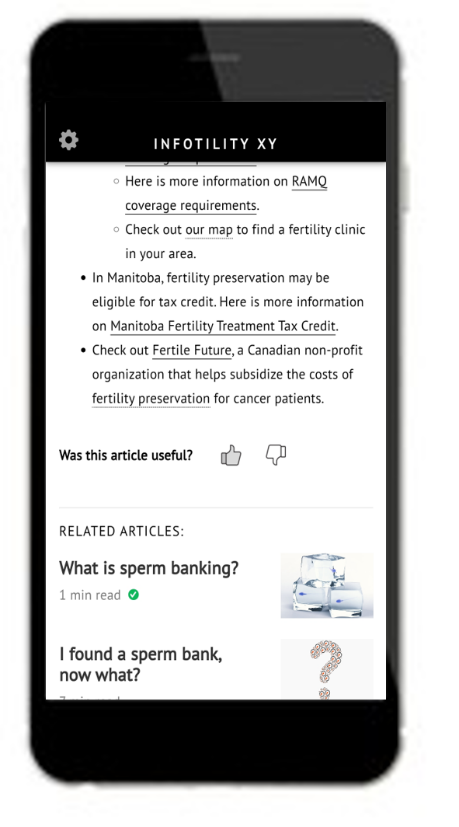


(A) (B) (C)

**Figure S3.** The article “Where should I go and how much will banking cost?”: the graphic and layout (A); the pop-up glossary definition for the term “fertility preservation” (B); and the option to give a thumbs up/thumbs down (C).


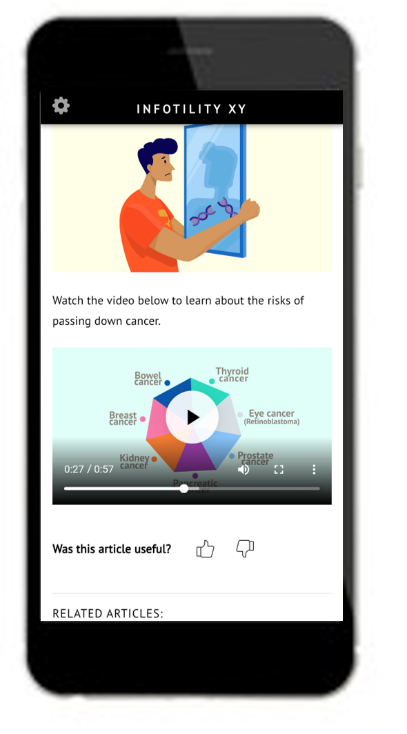

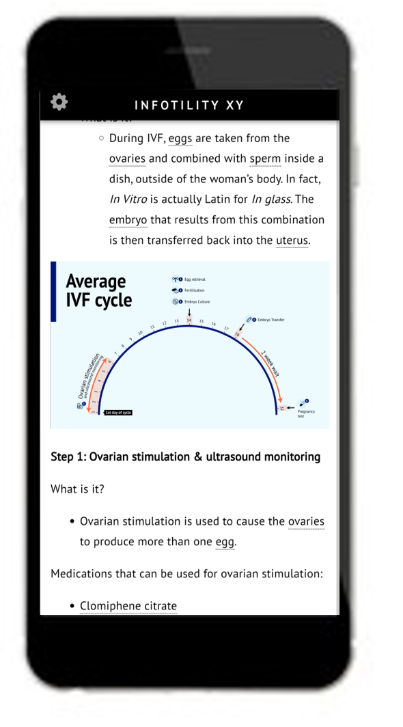


(A) (B)

**Figure S4.** The animation about the risks of passing down cancer in the article “I’ve heard that I can pass on cancer to my future child – is that true?” (A), and the infographic about in vitro fertilization in the article “Assisted Reproductive Technology (ART)” (B).

**
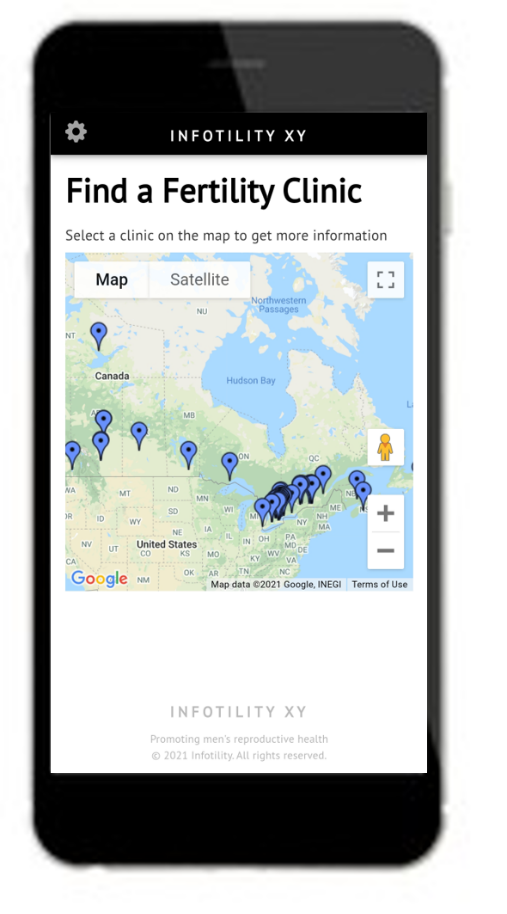

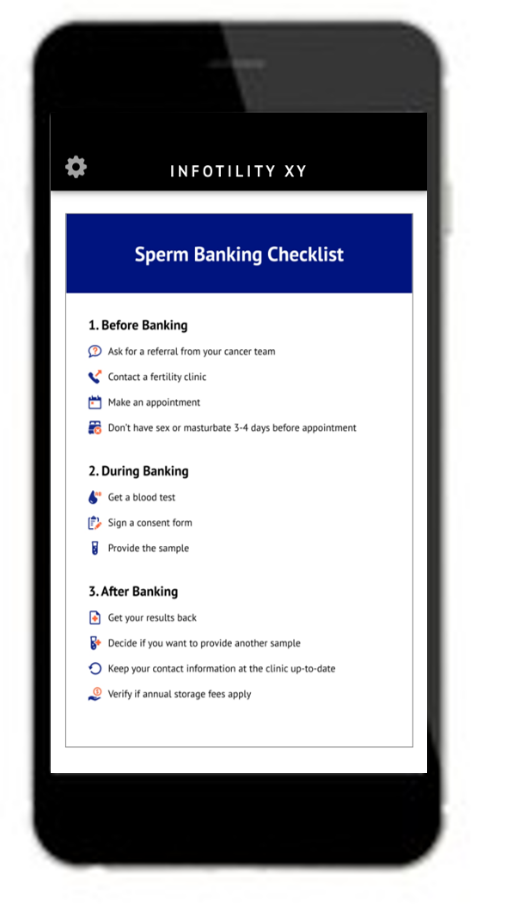
**

(A) (B)

**Figure S5.** The Canada-wide map of fertility clinics (A), and the infographic “Sperm Banking Checklist” (B).
