# Supplementary figures and images for: Evaluation of a Mobile Health App Offering Fertility Information to Male Patients With Cancer: Usability Study
Source: JMIR Cancer. 2022 May 4;8(2):e33594. doi: 10.2196/33594 (PMC9118008; doi:10.2196/33594)

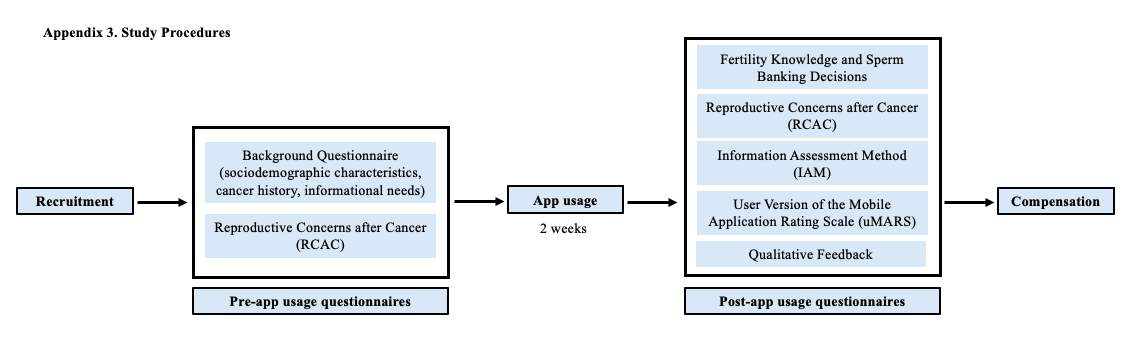

Supplement: Multimedia Appendix 3 [file cancer_v8i2e33594_app3.png]
