# Supplementary material for: Evaluation of a Mobile Health App Offering Fertility Information to Male Patients With Cancer: Usability Study
Source: JMIR Cancer. 2022 May 4;8(2):e33594. doi: 10.2196/33594 (PMC9118008; doi:10.2196/33594)
Supplement: Multimedia Appendix 4 [file cancer_v8i2e33594_app4.docx]

**Appendix 4.** Sample’s sociodemographic characteristics (N=40).

| Characteristics | Values, n (%) |
| --- | --- |
| **Relationship status** |  |
| Single | 8 (20) |
| In a heterosexual relationship | 27 (68) |
| In a nonheterosexual relationship | 5 (13) |
| **Do you have any children?** |  |
| Yes | 22 (55) |
| No | 18 (45) |
| **Would you like to have any children in the future?** |  |
| Yes | 33 (83) |
| No | 2 (5) |
| I don’t know | 5 (13) |
| **Born in Canada** |  |
| Yes | 35 (88) |
| No | 5 (13) |
| **Household income (CAD)** |  |
| Less than $50,000 | 4 (10) |
| $50,000- $89,999 | 19 (48) |
| $90,000 - $129,999 | 16 (40) |
| $130,000 and above | 1 (3) |
| **Highest education level** |  |
| Elementary | 0 |
| High school | 8 (20) |
| CEGEP^a^, trade, vocational | 15 (38) |
| Undergraduate/Bachelor’s degree | 13 (33) |
| Graduate or other professional degree | 4 (10) |
| **Ethnicity** |  |
| Indigenous | 1 (3) |
| White | 25 (63) |
| Black | 2 (5) |
| Latin, Central and South American | 4 (10) |
| Central Asian, North African and Middle Eastern origins | 3 (8) |
| East and Southeast Asian | 3 (8) |
| Mixed ethnicity | 2 (5) |
| **Do you consider yourself a religious person?** |  |
| Yes | 15 (38) |
| No | 25 (63) |

^a^CEGEP: a college preparatory program or technical program following high school in Quebec, Canada.
